# Integrative transcriptomic analysis deciphering the role of rice bHLH transcription factor Os04g0301500 in mediating responses to biotic and abiotic stresses

**DOI:** 10.3389/fpls.2023.1266242

**Published:** 2023-09-27

**Authors:** Qiuping Zhang, Rong Teng, Ziyi Yuan, Song Sheng, Yunhua Xiao, Huabing Deng, Wenbang Tang, Feng Wang

**Affiliations:** ^1^ College of Agronomy, Hunan Agricultural University, Changsha, China; ^2^ Yuelushan Laboratory, Changsha, China; ^3^ College of Forest, Central South University of Forestry and Technology, Changsha, China; ^4^ Hunan Hybrid Rice Centre, Hunan Academy of Agricultural Science, Changsha, China

**Keywords:** integrative transcriptomic analysis, rice, multi-step homolog search, OsbHLHs, biotic and abiotic stress

## Abstract

Understanding the signaling pathways activated in response to these combined stresses and their crosstalk is crucial to breeding crop varieties with dual or multiple tolerances. However, most studies to date have predominantly focused on individual stress factors, leaving a significant gap in understanding plant responses to combined biotic and abiotic stresses. The bHLH family plays a multifaceted regulatory role in plant response to both abiotic and biotic stresses. In order to comprehensively identify and analyze the bHLH gene family in rice, we identified putative OsbHLHs by multi-step homolog search, and phylogenic analysis, molecular weights, isoelectric points, conserved domain screening were processed using MEGAX version 10.2.6. Following, integrative transcriptome analysis using 6 RNA-seq data including Xoo infection, heat, and cold stress was processed. The results showed that 106 OsbHLHs were identified and clustered into 17 clades. Os04g0301500 and Os04g0489600 are potential negative regulators of Xoo resistance in rice. In addition, Os04g0301500 was involved in non-freezing temperatures (around 4°C) but not to 10°C cold stresses, suggesting a complex interplay with temperature signaling pathways. The study concludes that Os04g0301500 may play a crucial role in integrating biotic and abiotic stress responses in rice, potentially serving as a key regulator of plant resilience under changing environmental conditions, which could be important for further multiple stresses enhancement and molecular breeding through genetic engineering in rice.

## 1 Introduction

Environment changes induced by unfavorable growth conditions inevitably affect crop yield every year. Indeed, under long-term natural selection pressures, sessile plants have evolved a complex set of molecular and physiological mechanisms to cope with abiotic/biotic stresses. However, poverty, climate change, population growth, the COVID-19 pandemic, and even other potential risk forces agriculture system to evolve in a high-production and sustainable path. To adequately address the food security challenge, plant breeders expanding numerous tools and mining molecular resources to develop new improved crop varieties (Lenaerts et al., 2019; Zaidi et al., 2019). Moreover, the current emergence of the CRISPR-Cas systems in many crops provides an effective and straightforward suite to precisely and efficiently process genome editing (Zaidi et al., 2019). Precise genome editing requires an in-depth understanding of the underlying mechanisms with desired breeding goals.

Rice (*Oryza sativa* L.) stands as a cornerstone in global agriculture, serving as a staple food for a significant proportion of the world’s population. However, this crucial crop encounters a multitude of threats from both biotic and abiotic stresses. Among the major concerns is *bacterial blight* (BB), incited by the gram-negative bacterial pathogen *Xanthomonas oryzae pv. Oryza* (Xoo), and temperature-related stresses, including heat and cold stresses (Khush, 2005). Deciphering the intricate signaling pathways that rice employs in response to these concurrent stresses, and their intricate crosstalk, is pivotal for developing crop varieties with enhanced tolerance to these combined stresses. Atkinson and Urwin (2012) emphasized the need for more holistic approaches to plant stress research, considering the combined effects of multiple stress factors that crops face in the field, as opposed to studying individual stresses in isolation (Atkinson and Urwin, 2012). In *Arabidopsis*, the involvement of the *NPR1* (Nonexpresser of Pathogenesis-Related Genes 1) gene in combined stress responses has been highlighted. *NPR1*, a key regulator in salicylic acid-mediated resistance to pathogens, also confers tolerance to drought stress, signifying its role in the crosstalk between biotic and abiotic stress signaling pathways (Zhang et al., 2019). However, the majority of existing research has been centered on individual stress responses, leaving the cumulative impact of combined stressors somewhat understudied.

*bHLH* (basic Helix-Loop-Helix) transcription factors constitute the second largest families after the *MYBs* in plants and are involved in a broad range of biological processes. This group of proteins modulates both disease resistance and temperature stress tolerance. The *bHLH* transcription factors *PIF4* and *PIF5*, for example, are known to mediate plant responses to high temperatures by influencing auxin signaling pathways (Leivar and Quail, 2011). Conversely, the bHLH transcription factor ICE1 is documented to govern the CBF pathway, subsequently enhancing cold tolerance in plants (Chinnusamy et al., 2007). The functionality of bHLH transcription factors under the influence of combined temperature stresses, and potentially in conjunction with biotic stress such as bacterial blight, is an area that necessitates further exploration.

Indeed, extensive research of basic helix-loop-helix (bHLH) transcription factors in Oryza sativa has delineated the function of key members such as OsIRO2, OsbHLH006, and OsbHLH062, each of which has integral roles in iron homeostasis, abiotic stress responses, and disease resistance, respectively (Ogo, 2006; Seo et al., 2011; Liu et al., 2015). However, given the considerable number and diversity of bHLH transcription factors in rice, it is evident that a vast array of these proteins remains functionally uncharacterized (Toledo-Ortiz et al., 2003; Pires and Dolan, 2010; Guo et al., 2021; Wang et al., 2023). The intricacy and multifariousness of bHLH proteins substantiate the likelihood of unveiling additional functional aspects, particularly in scenarios where biotic and abiotic stress responses intersect. Thus, we focused on the bHLH genes and explored the gene function based on large scales of expression profiles from RNA-seq data sets including Xoo infection and temperature stresses. Through this rigorous exploration, we were able to identify putative core bHLH genes that not only participate in distinct stress responses but may also serve as crucial mediators in the crosstalk between Xoo infection and temperature stress. The findings from this work provide a significant step forward in unraveling the complex network of stress response and signal transduction in rice.

## 2 Materials and methods

### 2.1 Homologs sequence identification and characterization of *bHLHs* in rice

To comprehensive identification and analysis of the *bHLH* gene family in rice, the HMM (Hidden Markov Model) profile PF00010 (*bHLH* domain) was retrieved from the Pfam database (https://pfam.sanger.ac.uk/, accessed on 1 January 2020) to identify the putative *OsbHLH* genes from previous BLAST filtered candidates. Basic parameters including molecular weight (MW) and isoelectric point (pI) were predicted using the ProtParam tool (https://web.expasy.org/protparam/, accessed on 1 January 2020).

### 2.2 Phylogenetic and conserved motifs analysis of *OsbHLH* proteins

To analyze the sequence features of *OsbHLH* proteins, a total of 106 *bHLH* proteins were analyzed using MEGAX version 10.2.6 (https://www.megasoftware.net) using default “MUSCLE” parameters for alignment, and the phylogenetic. The tree was then constructed with default “Neighbor-joining” algorithm parameters. “.nwk” phylogenetic trees were then visualized by iTol (https://itol.embl.de/) (Letunic and Bork, 2016). Conserved motifs of *OsbHLH* proteins were predicted using the MEME suite version 5.4.1 (https://meme-suite.org/meme/index.html) (Bailey et al., 2015), with default parameters except “motifs should find” set to 10.

### 2.3 Integrative transcriptome analysis of *OsbHLH* genes in response to Xoo infection, heat and cold stresses

The raw RNA sequencing data was obtained from NCBI Sequencing Read Archive (SRA, https://www.ncbi.nlm.nih.gov/sra) through SRA toolkits “prefetch” (version 2.8.0), PRJNA482466 is genotype CBB23 (JG30 + Xa23) after inoculation of PXO99A and P99M2, *PRJNA525987* for plants after PXO99A and PH infection in JG30 genotype, *PRJNA433094* is CBB23 and JG30 before and after PXO99A inoculation, *PRJNA314700* is rice plant in responsive to Xoo infection and heat stress, *PRJNA610422* and *PRJEB22031* for 4°C and 10°C cold stresses, respectively (Buti et al., 2018; Tariq et al., 2018; Tariq et al., 2019; Wang et al., 2019; Dossa et al., 2020; Pan et al., 2020).

Raw data (raw reads) of fastq format were firstly qualified with FastQC program (online available, https://www.bioinformatics.babraham.ac.uk/projects/fastqc/) for Q20, Q30, GC-content and sequence duplication level then followed with processed in Hisat2 version 2.2.1 (Kim et al., 2015) for read alignment to rice genome downloaded from online available Rice Genome Hub (https://rice-genome-hub.southgreen.fr/node/143/621). The reads were subjected to fragments per kilobase of transcript per million fragments mapped (FPKM) conversion to obtain the expression value of genes and transcripts. In-house R scripts were used to analyze gene expression and generate heat maps. The heat map was created using ggplot2, reshape2, and dplyr in R version 4.1.2.

## 3 Results

### 3.1 Genome-wide identification of bHLH genes in O. Sativa

**Figure 1:**
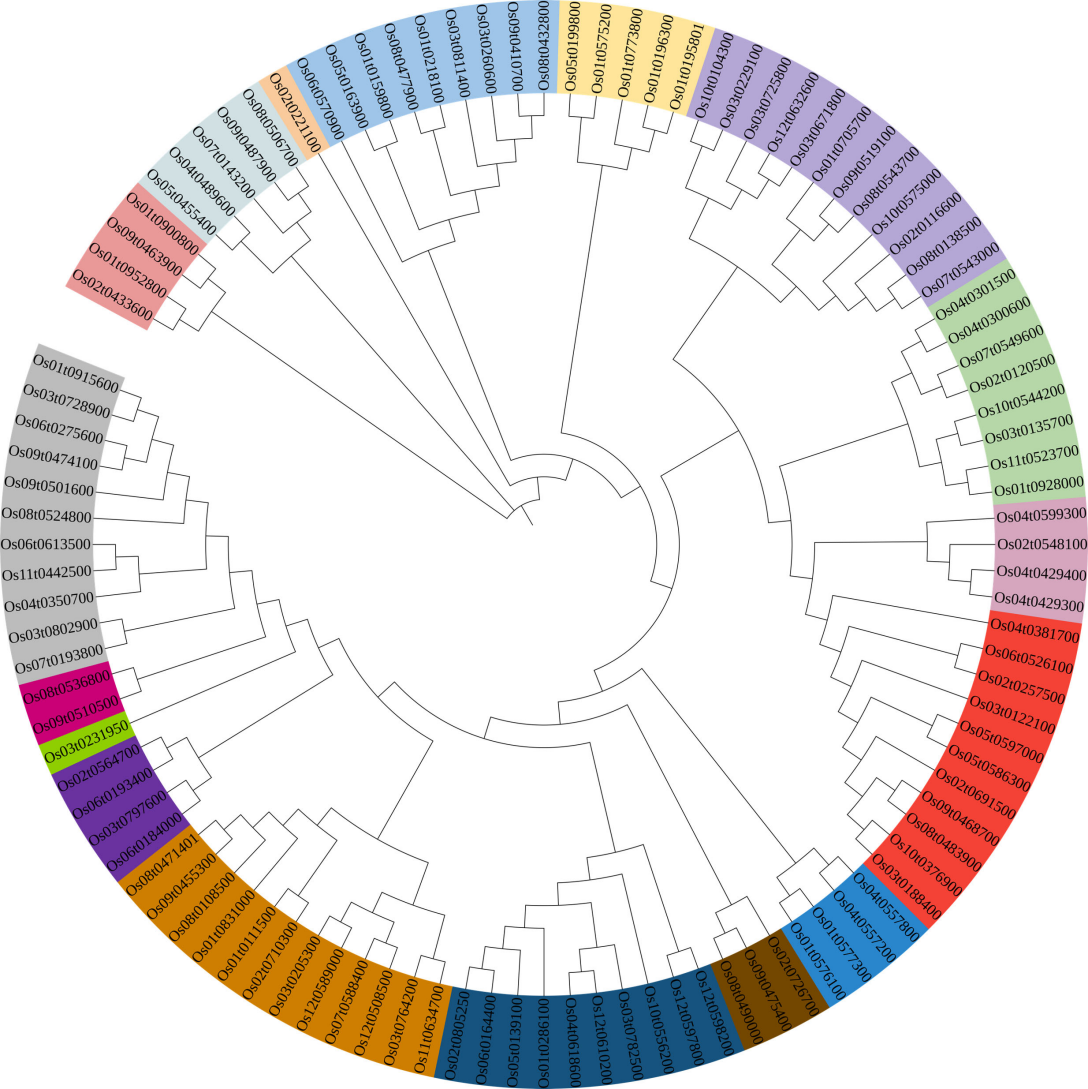
Phylogenetic tree of *bHLH* proteins in rice. Evolutionary relationships were constructed using the neighbor-joining (NJ) method. The shaded color indicates the different subfamily.

To identify the *OsbHLH* genes in rice, A total of 106 proteins containing *bHLH* domain(s) (PF00010) were originally obtained in the rice genome was identified via stepwise procedures, including local BLAST and HMM searches, which were processed at an E-value cutoff of 1E-5 (**Figure 1**). These *OsbHLH* genes range from 78 to 700 amino acids (average/median 346/339 aa).

### 3.2 Phylogenetic and conserved motifs analysis of OsbHLHs

**Figure 2:**
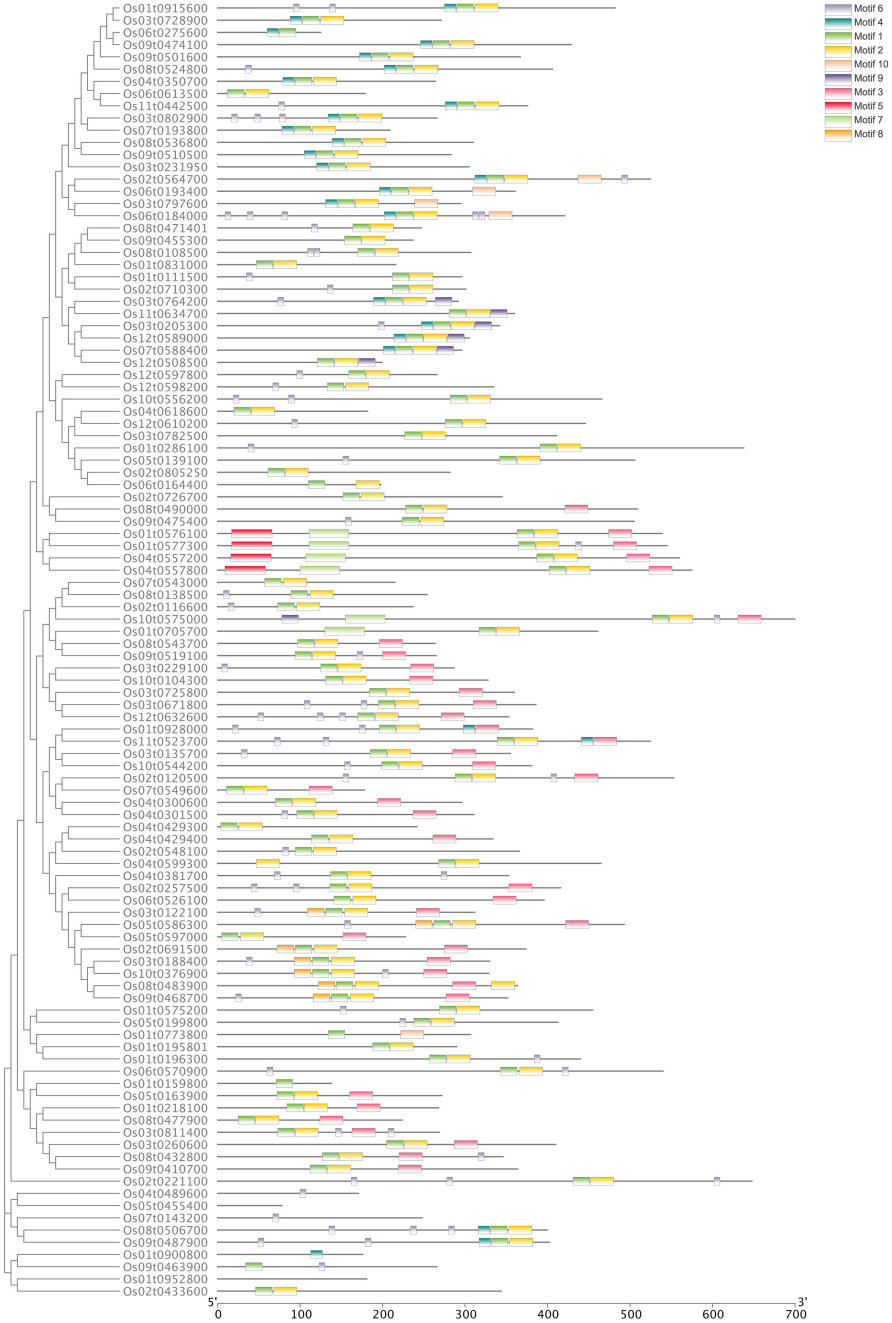
Analysis of the conserved motifs of OsbHLH proteins. Conserved motifs prediction is processed with MEME version 5.4.1.

A Neighbor-Joining phylogenetic tree was constructed based on 106 *OsbHLH* proteins that clustered into 17 clades (**Figure 1**). Furthermore, to explore the distribution and structural diversification of conserved motifs of *OsbHLH* proteins, we analyzed the conserved domains of *OsbHLHs* by the MEME (Multiple Em for Motif Elicitation) online tools (**Figure 2**). With only modifying the default setting of “motif numbers” from 3 to 10, we observed that none of the 106 *MsbHLHs* contain all 10 motifs while almost all proteins sharing the conserved *bHLH* domain (motif 2, yellow). The motif diversity was relatively conserved in each subfamily. In addition to motif 2, motifs 6 and 7 were also widely distributed.

### 3.3 Investigating the influence of TAL effectors on rice *bHLHs*

**Figure 3:**
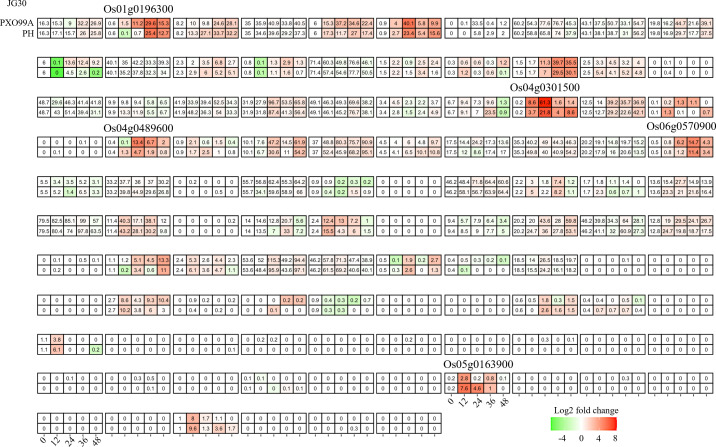
Candidate bHLHs selection in CBB23 after inoculation of PXO99A and P99M2 at different time points. Relative expression levels were calculated as a Log2-fold change against CK (see Section Materials and Methods). The red color shows an upregulation of a given gene, and the green indicates a downregulation. The labeled number in each tile is the expression level “FPKM”.

The *bHLH* family, one of the most diverse superfamilies, plays a multifaceted regulatory role in the plant response to both abiotic and biotic stresses. In the present study, our primary objective was to understand the impact of Xanthomonas oryzae pv. *Oryzae* (Xoo) infection on the transcriptome of rice. For this purpose, we analyzed RNA-seq data from a susceptible rice genotype, JG30, inoculated with both a wild Xoo strain, PXO99A, and its TAL effector-free mutant, PH. Time-course comparative analysis revealed that approximately 40% (40/106) of the *OsbHLH* genes showed altered expression following Xoo infection (**Figure 3**). However, a majority of these genes exhibited a similar response, indicating a generalized process of adaptation to Xoo, independent of TAL effector influence. We therefore shifted our attention to genes displaying differential regulation between infections caused by different Xoo strains. Six such genes, *Os01g0196300, Os01g0705700, Os04g0301500, Os04g0489600, Os06g0570900 and Os05g0163900*, were identified, potentially implying their involvement in the specific changes induced by TAL effectors and associated plant resistance.

### 3.4 Exploring the role of *bHLHs* in R gene-mediated resistance against Xoo

**Figure 4:**
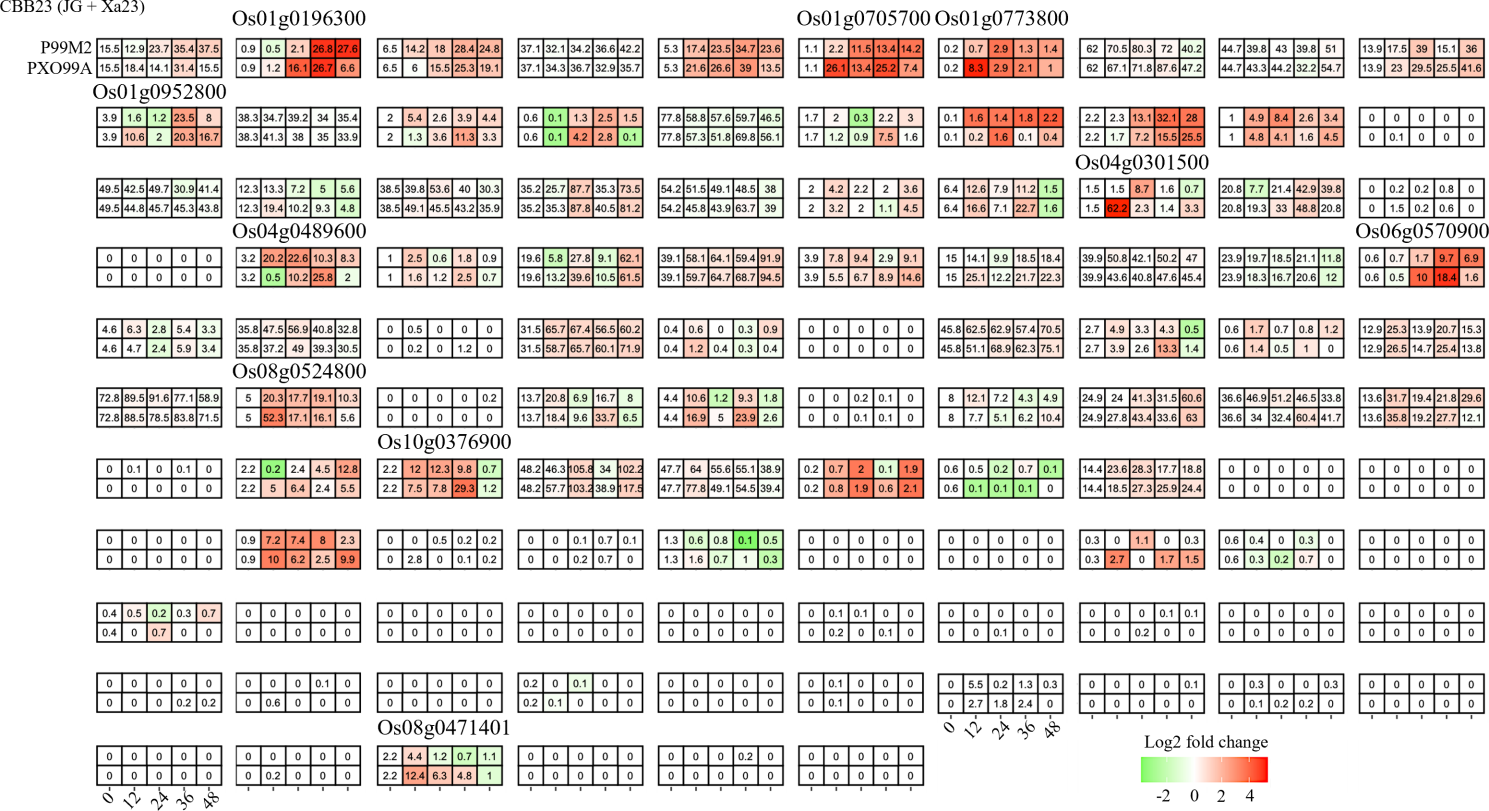
Candidate bHLHs selection at different time intervals after PXO99A and PH infection in the JG30 genotype. Relative expression levels were calculated as a Log2-fold change against CK (see Section Materials and Methods). The red color shows an upregulation of a given gene, and the green indicates a downregulation. The labeled number in each tile is the expression level “FPKM”.

To deepen our understanding of how Xoo exploits host susceptibility, we evaluated the RNA-seq data from the CBB23 line, harboring the Xa23 resistance gene, upon infection with the avrXa23-disrupted Xoo strain P99M2. Unlike in the previous set of experiments related to TAL effectors, here we found a larger set of *bHLHs* (52/106) responding to infection. Ten genes were identified that showed differential expression between infections caused by P99M2 and PXO99A (**Figure 4**). Interestingly, four of these genes were also differentially expressed in the prior study. This observation points towards potential overlap in the mechanisms of host response modulated by TAL effectors and R genes.

### 3.5 Cross-validation of *OsbHLH* genes associated with Xa23-mediated resistance

**Figure 5:**
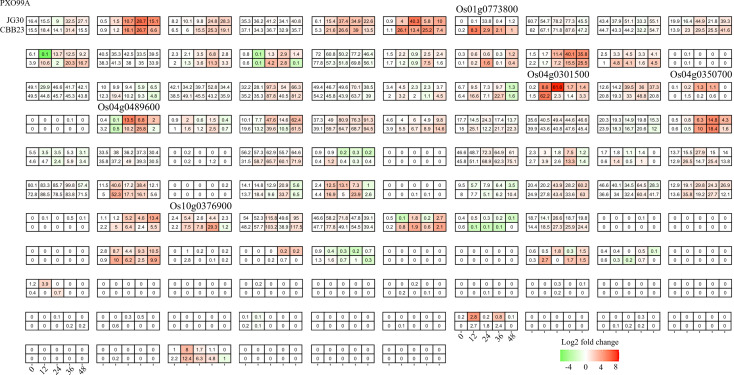
Candidate selection of differentially expressed responsive bHLHs in CBB23 and JG30 before and after PXO99A inoculation at different time points. Relative expression levels were calculated as a Log2-fold change against CK (see Section Materials and Methods). The red color shows an upregulation of a given gene, and the green indicates a downregulation. The labeled number in each tile is the expression level “FPKM”.

To further corroborate the role of *bHLH* genes in R gene-mediated responses to Xoo infection, we performed an integrated analysis of transcriptomic data from the near-isogenic lines (NILs) CBB23 (carrying Xa23) and JG30 (without Xa23), pre- and post-infection with the Xoo strain PXO99A. Nearly 45% of the *bHLHs* (47/106) displayed altered expression levels upon Xoo infection (**Figure 5**). Importantly, only two genes, *Os04g0301500* and *Os04g0489600*, matched those identified in the previous Xa23-related studies. Both these genes exhibited a consistent expression pattern across studies, indicating their potential as negative regulators of Xoo resistance in rice. Particularly noteworthy was the reduced expression of *Os04g0301500* in P99M2-infected CBB23, further supporting its role in mediating resistance responses.

### 3.6 Elucidating *Os04g0301500* and *Os04g0489600* in IRBB67 (Xa4 + Xa7) and response to temperature stress

**Figure 6:**
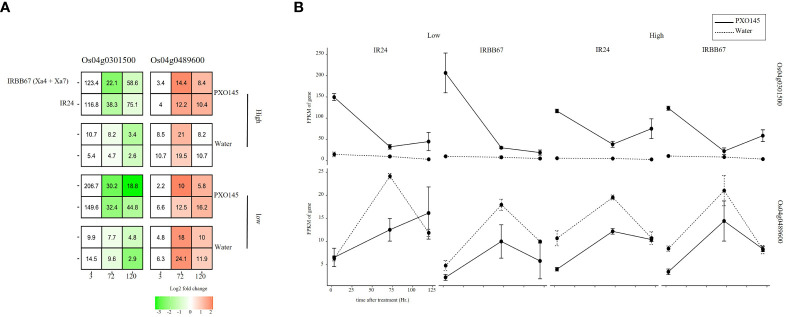
The expression of Os04g0301500 and Os04g0489600 in response to Xoo infection and heat stress. **(A)** heatmap of gene expression in response to combined stress. **(B)** Expression pattern of candidate genes.

The unique executor R gene, Xa23, confers an extremely robust, race-specific resistance against Xanthomonas oryzae pv. oryzae (Xoo) by encoding a small protein that, upon induction by the pathogen, initiates a process of cell death and thus resistance. To further elucidate the interplay between this resistance mechanism and the regulation of basic helix-loop-helix (*bHLH*) genes, we investigated the expression of two *bHLH* genes, *Os04g0301500* and *Os04g0489600*, under Xoo infection and different temperature conditions. Despite their general down- and up-regulation respectively when infected with Xoo strain PXO145, *Os04g0301500* exhibited a pronounced up-regulation in PXO145-infected tissues compared to its water-injected counterparts. This led us to corroborate its role as a negative regulator of Xoo resistance. The contrasting down-regulation of *Os04g0489600* in the same conditions, however, did not align with our prior assumptions(**Figure 6**). Moreover, upon comparing cultivars IRBB67 and IR24, a temperature-induced (29 to 35 °C) down-regulation of *Os04g0301500* was noted exclusively in IRBB67, hinting at a potential decreased tolerance to Xoo infection and absence of connection between the R genes Xa4 and Xa7 and the observed *bHLH* genes. Collectively, our findings consistently suggest a role for *Os04g0301500* in disease resistance and propose its potential crosstalk with temperature signaling pathways, shedding light on the complex dynamics of plant-pathogen interactions and stress adaptations in rice.

### 3.7 *Os04g0301500* in response to chilling and cold

**Figure 7:**
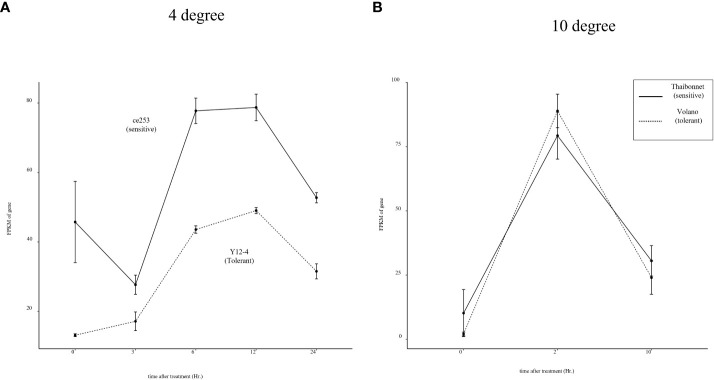
Expression pattern of Os04g0301500 in response to 4°C and 10°C cold stresses. **(A)** Expression pattern of Os04g0301500 in 4°C, **(B)** Expression pattern of Os04g0301500 in 10°C.

To broaden our comprehension of temperature-dependent stress responses, we expanded our scope to encompass data from RNA-seq experiments under chilling (10°C) and cold (4°C) stress conditions. Notably, as shown in **Figure 7A**, the expression of *Os04g0301500* was consistently higher in the sensitive cultivar Ce253 compared to Y12-4 and mirrored a similar induction pattern under cold stress. However, when subjected to chilling temperatures, no significant difference in gene expression was observed between sensitive and tolerant cultivars (**Figure 7B**). These findings suggest that the regulatory role of *Os04g0301500* might be specific to cold stress conditions rather than encompassing all temperature-related stresses. This specificity accentuates the complexity of stress response mechanisms in rice and underscores the necessity of more targeted analyses in future studies.

## 4 Discussion

The combined stresses from various environmental and pathogenic factors pose a substantial challenge to crop survival and productivity, necessitating in-depth comprehension of the underlying molecular mechanisms. Recent studies have shown that the response of plants to these combinations is unique and cannot be directly extrapolated from the response to individual stresses. The complexity of plant responses to stress combinations is largely controlled by different and sometimes opposing signaling pathways that may interact and inhibit each other, making it challenging to predict the impact on plant growth and physiological traits (Atkinson and Urwin, 2012; Suzuki et al., 2014). The basic helix-loop-helix (bHLH) superfamily, a diverse set of transcription factors, plays a pivotal role in mediating plant responses to these combined stresses (Heim, 2003; Pires and Dolan, 2010; Feller et al., 2011). Considering the complexity of Xoo infection in rice causing bacterial leaf blight (Niño-Liu et al., 2006). This bacterium employs various virulence factors, leading to a complex infection process (Büttner and Bonas, 2010). The plant immune system provides an intricate line of defense against such pathogens, contributing to the complexity of the interaction (Jones and Dangl, 2006). Given the potential role of *bHLH* transcription factors in plant stress responses, we decided to integrative analysis of RNA-seq from different perspectives to identify functional *bHLH* genes. In this study, we have incorporated three distinct RNA-seq datasets that provide a comprehensive perspective on the interaction between Xoo and rice. The datasets include scenarios with Xoo strains that are either without TAL effectors, or have the avrXa23 gene disrupted, and injected into CBB23 (JG30 + Xa23) cultivar, and its background cultivar JG30. TAL effectors, injected into the plant cells by Xoo, bind to specific plant genomic promoters, modulating gene expression in a way that often enhances conditions for bacterial prosperity and augments disease progression (Boch and Bonas, 2010). Contrastingly, plant R (resistance) genes play an instrumental role in the immune system by identifying and responding to specific pathogens or disease-causing agents (Jones and Dangl, 2006). Thus, by integrating these datasets, our analysis covers both pathogen-driven and host-mediated aspects of the bacterial blight (BB) disease, allowing for a deeper understanding of gene expression dynamics over time.

From the individual dataset, we identified 6, 10, 5 *bHLH* genes differentially regulated between treatment and control rice tissues (**Figures 3**-**5**). In general, these genes were induced in the relatively later phases of the infection experiments, most during 24–48 hp. Upon pathogen exposure, plants rapidly initiate a multi-tiered defense response to prevent pathogen invasion and propagation. The first line of defense, known as pathogen-associated molecular pattern-triggered immunity (PTI), is launched within minutes to hours of pathogen recognition, facilitated by pattern recognition receptors (PRRs) such as FLS2 and EFR that perceive specific molecular patterns on the pathogen surface fun (Boutrot and Zipfel, 2017). Signal perception and transduction trigger the activation of multiple downstream processes, including calcium influx, production of reactive oxygen species (ROS), activation of mitogen-activated protein kinase (MAPK) cascades, and induction of defense-related genes (Bigeard et al., 2015). Central to these early responses is the involvement of several transcription factors (TFs) that regulate the expression of defense-related genes. For example, *WRKY33*, a member of the *WRKY* family, is activated in the early stages of pathogen infection, mediating defense responses against necrotrophic pathogens in Arabidopsis through modulating the biosynthesis of phytoalexins and ROS homeostasis (Birkenbihl et al., 2012). Similarly, members of the *MYB*, bZIP, and NAC TF families, such as *MYB44*, *bZIP10*, *NAC019*, *NAC055*, and *NAC072*, are also rapidly induced upon infection, regulating various aspects of the defense response including stomatal closure, immune responses, cell death, and basal defense (Kaminaka et al., 2006; Jensen et al., 2008; Jung et al., 2008). Thus, we deduced that the differentially regulated *OsbHLH* may involve a later phase of the infection defense system, especially for the cross-validated *Os04g0301500* and *Os04g0489600*. These could include genes involved in hormone biosynthesis, signaling, and response, as well as genes involved in the synthesis of defensive compounds, cell wall reinforcement, and programmed cell death (Jones and Dangl, 2006).

To further verify the potent function of *Os04g0301500* and *Os04g0489600* involved in stress response, we examine transcriptomic data sourced from a near-isogenic line (NIL) IRBB67, which carries both Xa4 and Xa7 and its parental variety, IR24, under combined Xoo infection and temperature conditions. Interestingly, we observed no significant differential expression except for a notable temperature-induced down-regulation of *Os04g0301500* in IRBB67. It’s known that Xa7 imparts a durable and broad-spectrum resistance against bacterial blight disease in rice, showing enhanced efficacy under high-temperature conditions, while the effectiveness of other R genes decreases (Webb et al., 2010; Chen et al., 2021). Temperature improved the tolerance of Xoo in IRBB67. This could potentially clarify the observed downregulation of *Os04g0301500*, which we previously reported as a likely negative regulator, enhancing Xoo tolerance. Of note, Xa4 is present in almost all commercial indica hybrid rice varieties in China, while Xa3/Xa26 is distributed widely in both indica and japonica varieties (Hu et al., 2017; Deng et al., 2018). Encoded by Xa4 is a cell wall-associated kinase that confers race-specific resistance to Xoo throughout the rice growth stages by strengthening the cell wall, thereby preventing Xoo invasion (Sun et al., 2003; Afzal et al., 2008; Hu et al., 2017). However, it is plausible that these R genes do not interact with the *bHLH* genes. As for Xa23, the unique executor R gene, AvrXa23, has been discovered to be highly conserved across all tested Xoo isolates (Wang et al., 2014). This conservation might augment the virulence of Xoo, facilitating infection and proliferation within host plants. Furthermore, the widespread occurrence of AvrXa23 among natural Xoo strains might underpin the broad-spectrum resistance offered by Xa23 (Jiang et al., 2020). RNA-seq analysis corroborated the induction and suppressive capability of Xa23 concerning *Os04g0301500* (**Figure 4**). In summary, our findings suggest that *Os04g0301500* may play a significant role in Xoo tolerance and thermal stress response in rice plants.

**Figure 8:**
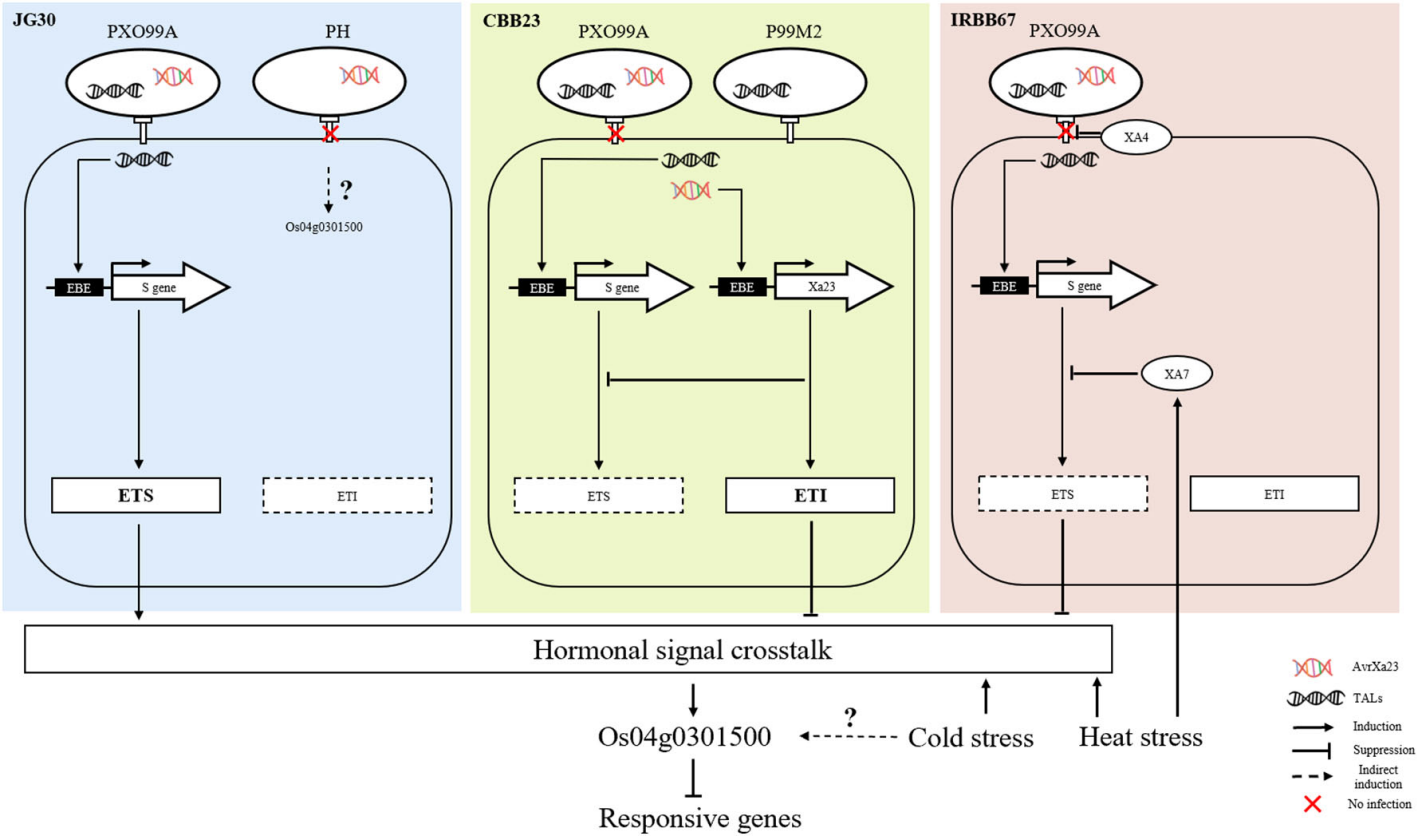
Summary of Os04g0301500 involved in combined stresses.

At the molecular level, the convergence of biotic and abiotic stress responses often involves hormonal cross-talk. Stress hormones like JA, salicylic acid (SA), and abscisic acid (ABA) regulate various aspects of plant defense and abiotic stress tolerance, and many TFs, including *bHLH* and WRKY proteins, are known to interact with these hormonal signaling pathways (Pieterse et al., 2009). One of the most well-documented examples of this phenomenon involves the SA-dependent pathway, typically activated in response to biotrophic and hemibiotrophic pathogens. Activation of R genes by these types of pathogens often leads to an increase in SA synthesis, which in turn triggers the expression of pathogenesis-related (PR) genes, contributing to localized and systemic acquired resistance (SAR) (Vlot et al., 2009). In rice, the *bHLH* transcription factor RERJ1 also reported involvement in wounding and drought response (Kiribuchi et al., 2005). To extend the understanding of *Os04g0301500* in stress response, transcriptome data of O. sativa subjected to chilling (10 °C) and cold (4°C) stress were analyzed. *Os04g0301500* only shows differentially regulated under cold between sensitive and tolerant rice cultivars. It is known that prolonged exposure to low non-freezing temperatures (around 4°C) can lead to cold acclimation, which increases freezing tolerance, and this involves the activation of CBF/DREB TFs. However the exact response of these TFs to a more moderate cold stress (such as 10°C) can depend on various factors including the plant species, the duration of the stress, and the plant’s developmental stage (Chinnusamy et al., 2007). As a result, we concluded that *Os04g0301500*, in addition to Xoo resistance and heat stress, may be implicated in cold acclimation (**Figure 8**).

## 5 Conclusion

In this study, our results suggest that the transcription factor *Os04g0301500* acts as a negative regulator of stress tolerance in response to bacterial blight (Xoo), as well as cold and heat stress in rice. We found that the expression of *Os04g0301500* was not significantly influenced by the resistance gene Xa4, but appeared to be modulated in coordination with Xa7 under increasing temperature conditions, which are known to enhance tolerance to Xoo. Our data also revealed that Xa4 is indirectly regulated by the resistance gene Xa23, through effector-triggered immunity and associated defense mechanisms. It’s well established that plant defense responses against pathogens involve a complex network of signal transduction pathways that integrate both biotic and abiotic stress signals to modulate plant immune responses. In this context, it appears that *Os04g0301500* might serve as a crucial node in this network, linking pathogen defense mechanisms with responses to abiotic stresses through hormonal signaling pathways. The precise mechanism through which *Os04g0301500* coordinates these responses remains to be elucidated, but it’s plausible that it might be involved in modulating the activity of hormonal signaling pathways in response to stress. For instance, ethylene, salicylic acid, and jasmonic acid are known to play critical roles in coordinating plant responses to both biotic and abiotic stresses. *Os04g0301500* could potentially influence the balance of these hormones, thereby fine-tuning the plant’s overall response to environmental stress. In conclusion, our findings highlight the potential role of *Os04g0301500* in integrating biotic and abiotic stress responses in rice, potentially serving as a key regulator of plant resilience under changing environmental conditions. Future research is warranted to further elucidate the precise role and underlying mechanisms of *Os04g0301500* in stress response modulation.

## Data availability statement

Publicly available datasets were analyzed in this study. This data can be found here: https://www.ncbi.nlm.nih.gov/sra (PRJNA482466, PRJNA525987, PRJNA433094, PRJNA314700, PRJNA610422 and PRJEB22031), https://rice-genome-hub.southgreen.fr/node/143/621.

## Author contributions

ZQ: Writing – original draft. TR: Data curation, Software, Writing – review & editing. YZ: Data curation, Writing – review & editing. SS: Data curation, Writing – review & editing, Formal Analysis. XY: Formal Analysis, Writing – review & editing. DH: Formal Analysis, Writing – review & editing. TW: Writing – review & editing, Project administration. WF: Writing – review & editing, Project administration, Data curation, Formal Analysis.
