# Supplementary figures and images for: Integrative transcriptomic analysis deciphering the role of rice bHLH transcription factor Os04g0301500 in mediating responses to biotic and abiotic stresses
Source: Front Plant Sci. 2023 Sep 27;14:1266242. doi: 10.3389/fpls.2023.1266242 (PMC10565216; doi:10.3389/fpls.2023.1266242)

# Supp. Figure 1

Os04g0301500

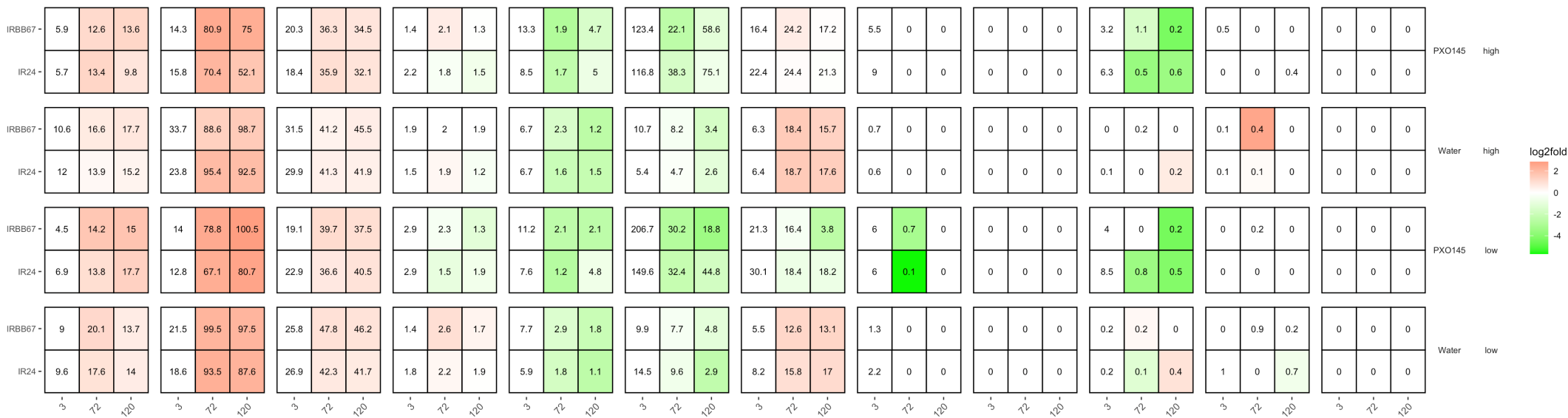

Os04g0489600

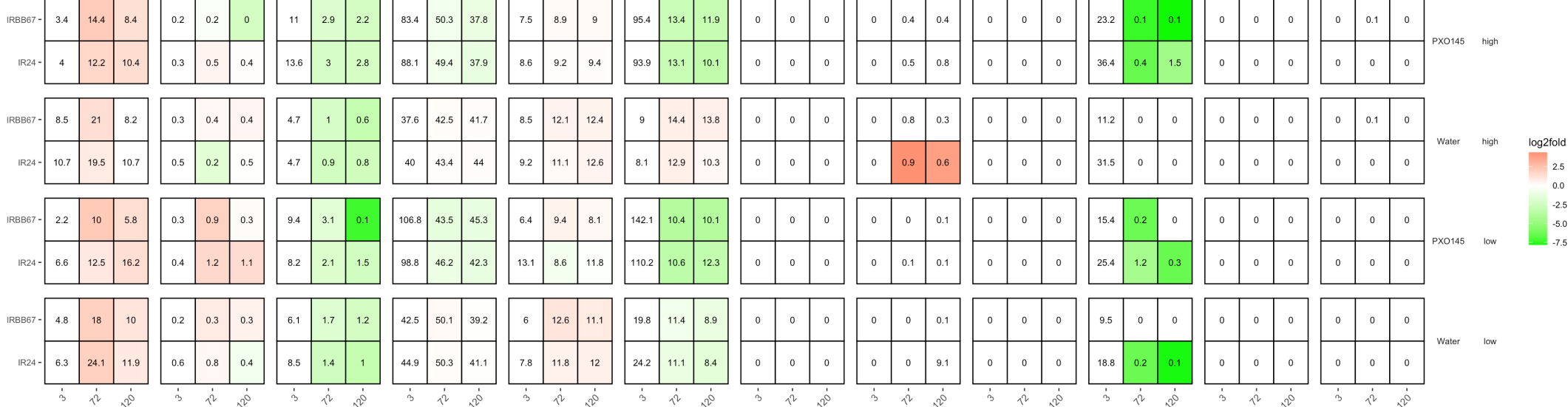

Supplement: Supplementary file 1 [file Image_1.pdf]
